# Correlation Between SII and Lymphocyte Subsets in Gastric Cancer Patients and Its Prognostic Importance

**DOI:** 10.1002/iid3.70399

**Published:** 2026-03-23

**Authors:** Xi Yan, Yinghao Niu, Jing Du, Gang Lu, Yiyang Guo, Jinyan Zhang, Yanyan Du, Xingxiao Yang, Ming Ma

**Affiliations:** ^1^ Department of Clinical Laboratory The Fourth Hospital of Hebei Medical University Shijiazhuang Hebei China; ^2^ Department of Clinical Biobank The First Hospital of Hebei Medical University Shijiazhuang Hebei China; ^3^ School of Medical Technologies Hebei Medical University Shijiazhuang Hebei China; ^4^ Department of Hospital Infection Management The Fourth Hospital of Hebei Medical University Shijiazhuang Hebei China

**Keywords:** biomarker, gastric cancer, lymphocyte subsets, prognosis, systemic immune‐inflammation index

## Abstract

**Purpose:**

This study aims to investigate the correlation and clinical significance of the systemic immune‐inflammation index (SII), which reflects the inflammatory state, with lymphocyte subsets representing the immune system.

**Methods:**

The clinical‐pathological and laboratory data of 95 patients with gastric cancer and 44 healthy controls were retrospectively analyzed in this study. The relationship between SII and clinicopathological characteristics, lymphocyte subsets, and other indicators was assessed through the Chi‐square test and Spearman correlation analysis. Kaplan‐Meier curves, log‐rank test, and Cox regression were used for survival analysis.

**Results:**

Compared with the control group, gastric cancer patients have higher SII (*p* < 0.01) and lower CD3^+^ T cell (*p* = 0.001), CD4^+^ T cell (*p* = 0.004), CD8^+^ T cell (*p* = 0.003), and B cell absolute counts (*p* < 0.01). Among them, SII has the highest diagnostic value for gastric cancer (AUC = 0.850, *p* < 0.01) and is positively correlated with TNM staging (*p* < 0.01). SII shows a significant negative correlation with the absolute counts of CD4^+^ T cells (r = −0.320, *p* = 0.002). Gastric cancer patients with high SII (*p* = 0.003) and low CD4^+^ T cell (*p* = 0.007), B cell (*p* = 0.001), and NK cell counts (*p* = 0.001) had shorter survival times. SII (HR = 3.304, *p* = 0.036) was independent risk factors influencing the prognosis of patients with gastric cancer.

**Conclusion:**

SII in gastric cancer patients exhibits the strongest correlation with CD4^+^ T cells among lymphocyte subsets, and an elevated SII is an independent risk factor for poor prognosis.

## 1 Introduction

Gastric cancer (GC) is associated with high morbidity and mortality, and although it has declined over the past decades, it remains one of the major health challenges worldwide [1]. The TNM staging system, a primary tool for staging GC, plays a critical role in guiding treatment strategies and predicting prognosis. However, the TNM classification system does not fully consider the tumor's biological behavior and the patient's immune‐inflammatory status, potentially impacting its precision in guiding treatment strategies and assessing prognosis. Tumor occurrence depends not only on the individual characteristics of the tumor, but also is affected by the systemic immune‐inflammatory response of the body [2, 3]. Inflammatory markers like the neutrophil‐lymphocyte ratio (NLR) and the monocyte‐lymphocyte ratio (MLR) are regarded as significant prognostic indicators for cancer [4, 5, 6, 7]. The systemic immune‐inflammatory response is pivotal in tumor development. The systemic immune‐inflammation index (SII) integrates neutrophil, platelet, and lymphocyte counts, offering a comprehensive reflection of the balance between host immune status and inflammation levels, with a notable advantage in predicting tumor prognosis [8, 9, 10].

Immunity and inflammation are the two core components of the tumor microenvironment, and immune response is also crucial in GC prognosis. As the main effector cells of the cellular immune response in the human body, lymphocytes reflect the status of the tumor microenvironment and may affect the prognosis of tumor patients [11, 12, 13]. Lymphocyte subsets include CD4^+^ T lymphocytes, CD8^+^ T lymphocytes, NK cells, and B cells. Presently, the connection between SII, lymphocyte subgroups, and how they collectively impact GC patient prognosis remains incompletely understood. Therefore, this study aims to investigate the association between SII and diverse lymphocyte subgroups and assess their clinical significance in predicting GC patient prognosis, providing more precise evidence for clinical treatment strategies.

## 2 Materials and Methods

### 2.1 Patients

We retrospectively analyzed 95 patients diagnosed with GC in the Fourth Hospital of Hebei Medical University from June 2019 to February 2023 as the GC group. These patients were included as the GC group. Inclusion criteria: (1) confirmed diagnosis of GC through gastroscopic biopsy; (2) complete clinical and pathological data available; (3) no prior neoadjuvant chemotherapy or any antitumor treatment; and (4) no infection or acute or chronic inflammatory response. Exclusion criteria: (1) patients who received GC‐related surgery or drugs; (2) concurrent history of other tumors; (3) severe infection (including systemic inflammatory response syndrome, sepsis, septic shock, or multiple organ dysfunction syndrome) or current use of immunosuppressive therapy; and (4) incomplete laboratory data. We selected 44 individuals from the same time frame who underwent regular health examinations at our hospital's health examination center and were ultimately classified as healthy for the control group. The inclusion criteria were as follows: (1) age and gender matched with the case group; (2) no known history of chronic diseases (such as hypertension, diabetes, cardiovascular and cerebrovascular diseases, tumors, etc.); (3) all health examination results within the normal reference range; and (4) no infections, autoimmune diseases, or other acute illnesses. This study adheres to the principles of the “Helsinki Declaration“ and was approved by the Medical Ethics Committee of the Fourth Hospital of Hebei Medical University (approval no: 2021KS037). Informed consent was waived by the Medical Ethics Committee of the Fourth Hospital of Hebei Medical University since it was a retrospective study.

### 2.2 Flow Cytometry

Lymphocyte subsets were analyzed using a four‐color antibody reagent kit (anti‐CD45/CD4/CD8/CD3, anti‐CD45/CD56/CD19/CD3) and were subjected to single‐platform detection using flow cytometry (Navios, Beckman Coulter, USA). Kaluza Analysis software was used to analyze the percentages and absolute counts of CD3^+^ T cells (CD3^+^), CD4^+^ T cells (CD3^+^ CD4^+^), CD8^+^ T cells (CD3^+^ CD8^+^), NK cells (CD3^−^ CD16^+^ CD56^+^), and B cells (CD3^−^ CD19^+^). The four‐color antibodies, Lysing Solution, and Flow‐Count Fluorospheres were all products of Beckman Coulter, USA.

### 2.3 Collection of Clinical and Laboratory Data and Calculation of Inflammatory Indexes

Patient characteristics (sex, age, primary tumor site, TNM stage and alcohol‐use status) were extracted from the institutional electronic medical‐record system, data had been documented in routine clinical practice at the time of initial admission. “Baseline levels” were defined as those generated from venous blood drawn within the first 24 h of hospital presentation, and no treatments, including antibiotics, NSAIDs, resection, radiotherapy, or chemotherapy, were administered before sample collection. Samples were processed in the Department of Clinical Laboratory on the day of collection. Complete blood counts were obtained with an automated haematology analyser (model BC‐6800 Plus, Mindray, China), yielding neutrophil, lymphocyte, platelet, and monocyte numbers. Serum tumor markers, include carbohydrate antigen 19‐9 (CA19‐9), CA72‐4 and carcinoembryonic antigen (CEA), were quantified by electrochemiluminescence immunoassay (Cobas e602, Roche, Germany). Peripheral‐blood lymphocyte subsets were determined with a six‐colour flow‐cytometry panel (Navios, Beckman, USA) and expressed as both percentages and absolute counts. The experimental procedures were strictly conducted according to standard operating procedures and the corresponding reagent manuals. Inflammatory indices were computed with the following formulas: SII = (neutrophil count × platelet count)/lymphocyte count; NLR = (neutrophil count/lymphocyte count); MLR = (Monocyte count/lymphocyte count).

### 2.4 Follow‐Up

All patients were followed up, primarily by reviewing their hospital or outpatient records and contacting them via phone. The follow‐up period extended until either July 2023 or the death of the patient. If there was no endpoint event or loss to follow‐up, the latest follow‐up status and time were to be treated as censored data. Overall survival (OS) was defined as the duration between the diagnosis of GC and either death or the last follow‐up date.

### 2.5 Statistical Analysis

SPSS 25.0 was used for statistical analysis and processing, and data visualizations were created using Prism version 8.0. Normality of all continuous variables was assessed using the Shapiro‐Wilk test, and continuous variables with non‐normal distribution were represented as the medians (interquartile ranges) [M (P25, P75)]. Since the data in this study were non‐normally distributed, the assumptions of parametric tests were violated. Therefore, the Mann‐Whitney *U* test was used for comparison between the two groups, and the Kruskal‐Wallis test was used for multiple group comparisons. Counts (percentage) represented the categorical data, and Chi‐square test was used for comparison among groups. Spearman correlation analysis was conducted to examine the relationships between variables, as this non‐parametric method does not require assumptions of normal distribution or linearity. Receiver operating characteristic (ROC) curve analysis was used to analyze the predictive value of each variable for GC. Optimal cut‐off values for continuous biomarkers were determined by maximizing the Youden index, which provides the point on the ROC curve that best balances sensitivity and specificity. Kaplan‐Meier method was used to analyze the relationship between variables and survival prognosis of GC, and Log‐Rank test was used for the comparison of survival. Univariate and multivariate Cox regression analysis were performed to identify risk factors influencing the prognosis of GC. The selection of variables included in the Cox regression model was guided by the following criteria: (1) statistical significance in univariate analysis and (2) absence of multicollinearity with other variables. The threshold for a statistically significant difference was 0.05.

## 3 Results

### 3.1 Comparison of the Demographic Characteristics and Baseline Laboratory Data of the Subjects

The median age of the 95 patients with GC was 64 years (range: 32–81), including 63 males (66.3%) and 32 females (33.7%). The median age of the 44 controls was 62.5 years (range: 50–79), including 23 males (52.3%) and 21 females (47.7%). There were no statistically significant differences in sex or age observed between the two groups (*p* > 0.05). Refer to Table 1 for the demographic characteristics and baseline laboratory data. The GC group exhibited significantly higher SII, NLR, MLR, CEA, CA19‐9, CA72‐4, and NK cell percentages than the control group (*p* < 0.05). While the absolute counts of CD3^+^ T cells, CD4^+^ T cells, CD8^+^ T cells, and B cells were significantly lower (*p* < 0.05). In contrast, there were no significant differences in the percentages of CD3^+^ T cells, CD4^+^ T cells, CD8^+^ T cells, CD4/CD8 ratio, or NK cell absolute count (*p* > 0.05).

**Table 1:** Demographic characteristics and baseline laboratory data in patient with gastric cancer and healthy control.

Clinical parameters	Control (*n *= 44)	gastric cancer (*n* = 95)	*χ* ^*2*^ */Z* value	*p* value
Gender [number (%)]				
Male	23 (52.3%)	63 (66.3%)	2.514	0.113
Female	21 (47.7%)	32 (33.7%)		
Age [number (%)]				
≤ 60	16 (36.4%)	34 (35.8%)	0.004	0.948
> 60	28 (63.6%)	61 (64.2%)		
Clinical data [median (P25, P75)]				
SII	370.88 (295.81, 432.04)	735.59 (503.39, 1202.87)	−6.625	< 0.001
NLR	1.72 (1.33, 1.94)	2.75 (2.09, 6.06)	−6.276	< 0.001
MLR	0.17 (0.13, 0.21)	0.27 (0.19, 0.41)	−5.312	< 0.001
CD3^+^ T cell (%)	73.58 (68.69, 76.09)	70.92 (64.45, 77.64)	−1.109	0.267
CD3^+^ T cell (/μL)	975 (778, 1187)	816 (542, 1023)	−3.380	0.001
CD4^+^ T cell (%)	40.59 (36.41, 46.27)	39.34 (32.81, 44.72)	−1.302	0.193
CD4^+^ T cell (/μL)	568 (427, 669)	445 (297, 594)	−2.876	0.004
CD8^+^ T cell (%)	25.50 (22.64, 34.31)	25.83 (20.32, 32.24)	−0.657	0.511
CD8^+^ T cell (/μL)	379 (250, 479)	265 (179, 402)	−2.968	0.003
CD4/CD8 ratio	1.54 (1.07, 1.96)	1.47 (1.08, 2.18)	−0.138	0.890
B cell (%)	8.91 (7.21, 11.85)	6.83 (4.13, 11.45)	−2.540	0.011
B cell (/μL)	128 (84, 186)	82 (42, 119)	−3.711	< 0.001
NK cell (%)	14.87 (12.43, 17.80)	17.68 (12.78, 24.19)	−2.602	0.009
NK cell (/μL)	198 (153, 256)	214 (91, 332)	−0.174	0.862
CEA (ng/mL)	1.86 (1.32, 2.74)	3.33 (1.95, 13.33)	−4.021	< 0.001
CA19‐9 (U/mL)	8.91 (6.39, 14.68)	17.01 (7.92, 66.28)	−3.557	< 0.001
CA72‐4 (U/mL)	2.15 (1.12, 4.69)	3.24 (1.46, 17.91)	−2.117	0.034

*Note:* The median (P25, P75) is a statistical measure used to describe the central tendency and dispersion of quantitative data, where P25 represents the 25th percentile (lower quartile), and P75 represents the 75th percentile (upper quartile).

### 3.2 Diagnostic Effectiveness of SII and Lymphocyte Subsets in Gastric Cancer

**Figure 1:**
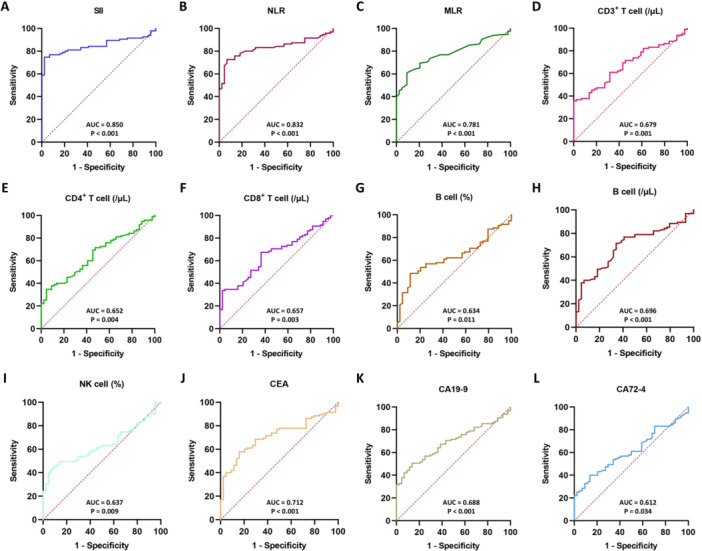
ROC curves for predicting gastric cancer. ROC curve analysis was performed to evaluate the diagnostic performance of individual biomarkers. (A) SII, systemic immune‐inflammation index; (B) NLR, neutrophil‐lymphocyte ratio; (C) MLR, monocyte‐lymphocyte ratio; (D) CD3^+^ T cell (/μL), absolute count of CD3^+^ T cells; (E) CD4^+^ T cell (/μL), absolute count of CD4^+^ T cells; (F) CD8^+^ T cell (/μL), absolute count of CD8^+^ T cells; (G) B cell (%), percentage of B cells; (H) B cell (/μL), absolute count of B cells; (I) NK cell (%), percentage of NK cells; (J) CEA, carcinoembryonic antigen; (K) CA19‐9, serum carbohydrate antigen 19‐9; (L) CA72‐4, serum carbohydrate antigen 72‐4. AUC with *p* values is shown for each predictor.

We next conducted ROC analysis on three systemic inflammatory indices, six lymphocyte subset parameters, and three conventional tumor markers. The results indicated that, among the evaluated indices, SII showed the largest AUC value (0.850; 95% CI: 0.786–0.914; *p* < 0.001) and may thus offer some auxiliary value for distinguishing gastric‐cancer patients from healthy controls. The sensitivity and specificity of SII were 74.70% and 97.70%, outperforming other indicators, such as NLR (AUC = 0.832, 95% CI: 0.764–0.899), MLR (AUC = 0.781, 95% CI: 0.706–0.855), CEA (AUC = 0.712, 95% CI: 0.628–0.797), CA19‐9 (AUC = 0.688, 95% CI: 0.519–0.705), and CA72‐4 (AUC = 0.612, 95% CI: 0.519–0.705). The lymphocyte subset‐related markers showed diagnostic value for GC with AUC values above 0.6, but lower sensitivity and specificity. Refer to Table 2 and Figure 1 for further details. Using Youden's index, the optimal cutoff values for SII, CD3^+^ T cell count, CD4^+^ T cell count, CD8^+^ T cell count, B cell percentage, B cell count, and NK cell percentage were determined as follows: 511.97, 632/μL, 367/μL, 210/μL, 6.36%, 111/μL, and 19.62%, respectively.

**Table 2:** Predictive efficacy of SII and lymphocyte subsets in predicting gastric cancer.

Indicator	AUC (95% CI)	*p* value	Sensitivity (%)	Specificity (%)	Youden's index (%)
SII	0.850 (0.786–0.914)	< 0.001	74.70	97.7	72.40
NLR	0.832 (0.764–0.899)	< 0.001	72.6	93.2	65.80
MLR	0.781 (0.706–0.855)	< 0.001	63.2	90.9	54.1
CD3^+^ T cell (/μL)	0.679 (0.591–0.766)	0.001	35.80	93.20	35.80
CD4^+^ T cell (/μL)	0.652 (0.561–0.743)	0.004	34.70	95.50	30.20
CD8^+^ T cell (/μL)	0.657 (0.565–0.748)	0.003	33.70	63.60	20.95
B cell (%)	0.634 (0.542–0.726)	0.011	48.40	88.60	37.00
B cell (/μL)	0.696 (0.608–0.784)	< 0.001	71.60	65.90	37.50
NK cell (%)	0.637 (0.547–0.727)	0.009	43.20	93.20	36.40
CEA (ng/mL)	0.712 (0.628–0.797)	< 0.001	57.90	84.10	42.00
CA19‐9 (U/mL)	0.688 (0.519–0.705)	< 0.001	50.50	86.40	36.90
CA72‐4 (U/mL)	0.612 (0.519–0.705)	0.034	40.00	86.40	26.40

### 3.3 The Correlation Between SII and Clinicopathological Characteristics of Gastric Cancer

GC patients were categorized into low SII (SII ≤ 511.97) and high SII (SII > 511.97) groups, consisting of 24 and 71 cases, respectively. The results revealed a correlation between baseline SII levels and T stage (*p* = 0.002), N stage (*p* = 0.040), M stage (*p* = 0.032), and TNM stage (*p* < 0.001). However, no correlation was found with sex (*p* = 0.647), age (*p* = 0.077), tumor site (*p* = 0.424), or history of alcohol consumption (*p* = 0.374). For further details, please consult Table 3.

**Table 3:** The relationship between SII and clinical pathological features.

Parameters	*n*	SII ≤ 511.97 (*n* = 24)	SII > 511.97 (*n* = 71)	*χ* ^*2*^ value	*p* value
Gender				0.209	0.647
Male	63	15 (62.5%)	48 (67.6%)		
Female	32	9 (37.5%)	23 (32.4%)		
Age				3.126	0.077
≤ 60	34	5 (20.8%)	29 (40.8%)		
> 60	61	19 (79.2%)	42 (59.2%)		
Location				0.640	0.424
Cardia	37	11 (45.8%)	26 (36.6%)		
Non‐cardia	58	13 (54.2%)	45 (63.4%)		
Drinking history				0.791	0.374
Yes	56	16 (66.7%)	40 (56.3%)		
No	39	8 (33.3%)	31 (43.7%)		
Stage *T*				14.406	0.002
*T*_1_	4	2 (8.3%)	2 (2.8%)		
*T*_2_	11	7 (29.2%)	4 (5.6%)		
*T*_3_	10	4 (16.7%)	6 (8.5%)		
*T*_4_	70	11 (45.8%)	59 (83.1%)		
Stage N				8.311	0.040
N_0_	18	6 (25.0%)	12 (16.9%)		
N_1_	17	7 (29.2%)	10 (14.1%)		
N_2_	44	11 (45.8%)	33 (46.5%)		
N_3_	16	0 (0.0%)	16 (22.5%)		
Stage M				4.625	0.032
M0	62	20 (83.3%)	42 (59.2%)		
M1	33	4 (16.7%)	29 (40.8%)		
Stage TNM				20.530	< 0.001
Ⅰ–Ⅱ	18	12 (50.0%)	6 (8.5%)		
Ⅲ	44	8 (33.3%)	36 (50.7%)		
Ⅳ	33	4 (16.7%)	29 (40.8%)		

### 3.4 Correlation Between SII and Lymphocyte Subsets and Tumor Markers in Gastric Cancer Patients

**Figure 2:**
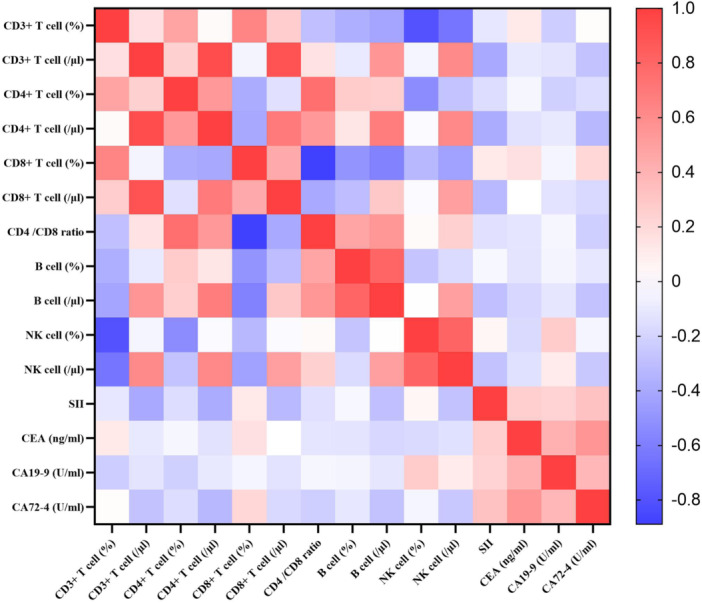
Correlation heatmap of SII, lymphocyte subpopulations, and tumor markers in gastric cancer patients. Spearman correlation analysis was performed to examine the relationships between variables. The heatmap displays correlation coefficients (r) ranging from −1.0 (blue, strong negative correlation) to + 1.0 (red, strong positive correlation). Variables include percentages and absolute counts of CD3^+^ T cells, CD4^+^ T cells, CD8^+^ T cells, B cells, and NK cells, as well as SII, CEA, CA19‐9, and CA72‐4.

A heatmap was plotted based on the results of Spearman correlation analysis to observe the correlations between the SII and 14 indicators. SII showed negative correlations with the counts of CD3^+^ T cell, CD4^+^ T cell, CD8^+^ T cell, B cell, and NK cell (*p* = 0.001, *p* = 0.002, *p* = 0.010, *p* = 0.020, *p* = 0.027) and a positive correlation with CA72‐4 (*p* = 0.013). Among these, the correlations with CD3^+^ T cell count and CD4^+^ T cell count (*r* = −0.331, *r* = −0.320) were relatively strong. However, no significant correlations were found between SII and the CD3 + T cells percentage, CD4 + T cells percentage, CD8 + T cells percentage, CD4/CD8 ratio, B cells percentage, NK cells percentage, CEA, or CA19‐9 (*p* = 0.389, *p* = 0.238, *p* = 0.395, *p* = 0.282, *p* = 0.787, *p* = 0.760, *p* = 0.053, *p* = 0.086), as illustrated in Figure 2.

### 3.5 The Distribution of SII and Absolute Lymphocyte Count in Clinical Stage of Gastric Cancer Patients

**Figure 3:**
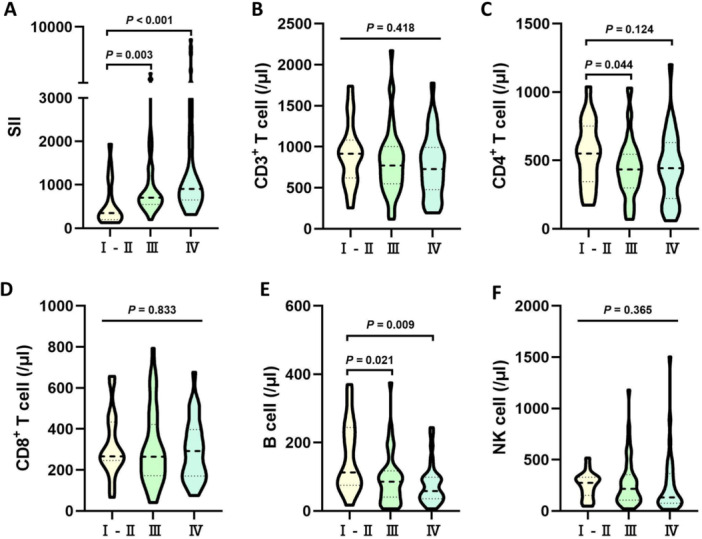
Distribution of SII and lymphocyte subpopulations in gastric cancer patients across different clinical stages. Violin plots show the distribution of (A) systemic immune‐inflammation index (SII), (B) absolute CD3^+^ T cell count (/μL), © absolute CD4^+^ T cell count (/μL), (D) absolute CD8^+^ T cell count (/μL), (E) absolute B cell count (/μL), and (F) absolute NK cell count (/μl) in patients with stage I ‐ II (*n* = 18), stage III (*n* = 44), and stage IV (*n* = 33) gastric cancer. Statistical comparisons between groups were performed using the Kruskal–Wallis test followed by Dunn's post‐hoc test for multiple comparisons.

The study findings revealed that compared to stages I–II, patients with stage III and IV GC had significantly elevated SII levels, with statistically significant differences (*p* = 0.003, *p* < 0.001). Moreover, the absolute counts of lymphocyte subsets, including CD3 + T cells, CD4 + T cells, B cells, and NK cells, demonstrated varying degrees of decline. Among these, CD4 + T cell counts exhibited a significant decrease in stage III patients (*p* = 0.044), and B cell counts showed significant reductions in both stage III and IV patients (*p* = 0.021, *p* = 0.009). However, there were no notable distinctions in the levels of SII, CD4 + T cells, and B cells among patients at stages III and IV (*p* > 0.05), as illustrated in Figure 3.

### 3.6 Correlation Between SII, Absolute Peripheral Blood Lymphocyte Count and Prognosis of Patients With Gastric Cancer

**Figure 4:**
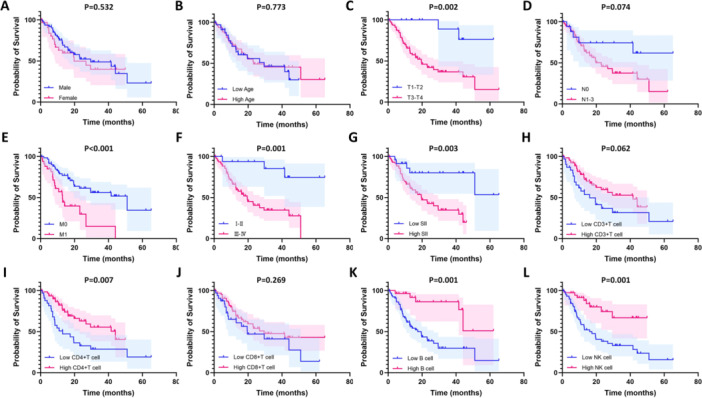
Kaplan–Meier survival curves for overall survival in gastric cancer patients stratified by diverse parameters. Overall survival was compared between groups using the Log‐rank test. (A) sex; (B) age; (C) T staging; (D) N staging; (E) M staging; (F) TNM staging; (G) systemic immune‐inflammation index (SII); (H) absolute CD3^+^ T cell count; (I) absolute CD4^+^ T cell count; (J) absolute CD8^+^ T cell count; (K) absolute B cell count; (L) absolute NK cell count. The optimal cut‐off values for continuous variables were determined using the Youden index. Shaded areas represent 95% confidence intervals.

A total of 44 deaths occurred in GC patients, resulting in an overall survival rate of 53.68%. After grouping the patients based on cutoff values, we further investigated the impact of various parameters on the overall GC survival rate. The results of Kaplan–Meier analysis indicated that earlier T stage (log‐rank = 9.331, *p* = 0.002), earlier M stage (log‐rank = 12.195, *p* < 0.001), and earlier TNM stage (log‐rank = 10.487, *p* = 0.001) had a longer survival. Additionally, there was a difference in the survival time between the high SII group and the SII group in GC patients, and the survival time of the high SII group was shorter (log‐rank = 9.124, *p* = 0.003). Furthermore, patients with lower absolute counts of CD4^+^ T cells (log‐rank = 7.159, *p* = 0.007), B cells (log‐rank = 12.071, *p* = 0.001), and NK cells (log‐rank = 11.448, *p* = 0.001) had shorter survival, as shown in Figure 4.

The proportional hazards assumption was assessed using Schoenfeld residual analysis. No significant violations were observed (*p* > 0.05), confirming the validity of the Cox regression results. Detailed test statistics are provided in Supporting Information Table 1. In the univariate Cox regression analysis, T stage, M stage, TNM stage, SII, CD4^+^ T cells, B cells, and NK cells were significantly associated with survival (*p* < 0.05), with the corresponding hazard ratios and 95% CIs presented in Table 4. Due to multicollinearity between TNM stage and T stage/M stage (variance inflation factor > 5), TNM stage was the only variable retained for multivariate analysis to avoid model instability. The multivariate Cox regression analysis revealed that SII (HR = 3.304, 95% CI: 1.084–10.073, *p* = 0.036) is an independent risk factor for overall survival in GC patients, while TNM stage, CD4^+^ T cells, B cells, and NK cells did not exhibit independent prognostic value (*p* > 0.05), as outlined in Table 4.

**Table 4:** Univariate and multivariate analysis for OS in gastric cancer patients.

	Univariate analysis			Multivariate analysis		
	*P*	*HR*	95% CI	*P*	*HR*	95% CI
Gender (male/female)	0.533	1.218	0.656–2.261	—	—	—
Age (≤ 60/ > 60)	0.774	0.914	0.495–1.688	—	—	—
T Staging (T_1‐2_/T_3‐4_)	0.008	6.823	1.642–28.349	—	—	—
N Staging (N_0_/N_1‐3_)	0.082	2.297	0.899–5.872	—	—	—
M Staging (M_0_/M_1_)	0.001	2.905	1.556–5.423	—	—	—
TNM Staging (Ⅰ‐Ⅱ/Ⅲ‐Ⅳ)	0.004	5.791	1.762–19.033	0.106	2.854	0.799–10.192
SII (≤ 511.97/ > 511.97)	0.005	4.341	1.540–12.236	0.036	3.304	1.084–10.073
CD3^+^ T cell (/μl) (≤ 632/ > 632)	0.065	0.565	0.308–1.037	—	—	—
CD4^+^ T cell (/μl) (≤ 367/ > 367)	0.009	0.451	0.247–0.821	0.082	0.541	0.270–1.081
CD8^+^ T cell (/μl) (≤ 210/ > 210)	0.272	0.710	0.385–1.308	—	—	—
B cell (/μl) (≤ 111/ > 111)	0.002	0.219	0.086–0.560	0.051	0.379	0.143–1.004
NK cell (/μl) (≤ 286/ > 286)	0.001	0.287	0.133–0.620	0.207	0.572	0.240–1.362

## 4 Discussions

The primary objective of this study is to identify easily accessible blood biomarkers that enhance the prognostic stratification of GC patients, thereby compensating for traditional TNM staging. While TNM staging serves as the cornerstone for predicting outcomes and guiding treatment, patients with the same stage often experience significantly different survival outcomes [14], partly due to TNM's inability to reflect the host's systemic immune‐inflammatory response. The immune‐inflammatory response is crucial for the prognosis of GC; any imbalance in this status within the host can promote the adhesion and distant metastasis of circulating tumor cells [15, 16]. SII, NLR, and MLR are indicators that evaluate the body's inflammatory status and have demonstrated clinical value in prognostic assessment and treatment efficacy prediction in cancers such as rectal [17], lung [18], and prostate cancer [19]. The quantity and percentage of lymphocyte subsets serve as important indicators of the body's immune functional status [20, 21]. This study retrospectively analyzed inflammatory indicators, characteristics of peripheral blood lymphocyte subsets, and classical serum tumor markers to explore the biological connections between pro‐tumor inflammation and anti‐tumor immunity, and to correlate these parameters with the prognosis of GC patients. The aim is to provide a simple and rapid tool that offers prognostic stratification benefits for personalized postoperative monitoring and treatment planning for these patients.

Initially, we examined the baseline characteristics of patients with GC and healthy individuals. The findings revealed a significant increase in SII, NLR, and MLR, representing systemic inflammation in GC patients, alongside markedly lower absolute counts of CD3 + T cells, CD4 + T cells, CD8 + T cells, and B cells, representing the immune system. These results suggest a disturbance in the immune‐inflammatory status in GC patients, and a microenvironment favoring tumor growth. We then used ROC curves to analyze the diagnostic efficacy of SII, NLR, MLR, lymphocyte subsets, and serum tumor markers in patients with GC. The results indicated that, compared to other markers, SII may provide some auxiliary value in distinguishing GC patients from healthy controls. However, it should not be considered a standalone diagnostic tool, and its clinical utility requires further validation in larger prospective cohorts. Further analysis of the factors influencing SII revealed that patients with GC at stage Ⅲ‐Ⅳ exhibited elevated SII levels, consistent with the study by Zhang [22], suggesting that SII is correlated with GC progression, has great potential as a prognostic marker of GC. The SII is determined using the lymphocyte count, yet the precise lymphocyte subpopulation that exhibits the highest correlation with SII remains uncertain. Therefore, we conducted correlation analysis, and the results indicated that the absolute count of diverse lymphocyte subpopulations, rather than their relative percentages, correlates with SII. This suggests that variations in the proportion of lymphocyte subpopulations do not impact the body's inflammatory status, whereas a reduction in the absolute count exacerbates the inflammatory response. Notably, the absolute count of CD4^+^ T cells exhibits the strongest association with SII. CD4^+^ T cells play a pivotal role in orchestrating anti‐tumor immune responses by recruiting and activating innate immune cells like NK cells and macrophages, demonstrating anti‐tumor functions, and secreting cytokines to stimulate humoral immunity [23, 24]. Our study reinforces the intimate connection between CD4^+^ T cells and inflammatory responses.

Kaplan–Meier survival analysis in this study showed that the SII (SII > 511.97) was the most effective predictor of overall survival in GC patients. This is consistent with the studies by Wang [25] and Hirahara [26]. Furthermore, Zhang et al. demonstrated that SII is an independent risk factor affecting the prognosis of GC patients, and it is also significantly correlates with OS of GC patients in different stages [27]. Additionally, there are literature reports indicating that patients with nasopharyngeal carcinoma with low CD19^+^ B cell or CD4^+^ T cell levels before treatment have a poorer prognosis [28]. Adequate circulating NK cells enable patients with metastatic colon cancer to achieve longer survival [29], and the characteristics of T cells, B cells, and Treg cells are independent predictors of postoperative recurrence in GC patients [30]. Our study confirmed that CD4^+^ T cell, B cell, and NK cell counts were higher in GC patients with long survival. Univariate and multivariate analyses showed that in addition to TNM staging, SII and CD4^+^ T cell count were independent risk factors for predicting overall survival in GC patients. SII, as a comprehensive indicator reflecting immune inflammation status, was inversely associated with CD4^+^ T cell count. We speculate that SII may affect tumor prognosis in the following ways: (1) neutrophils could participate in the reshaping of tumor immune microenvironment by releasing reactive oxygen species, growth factors, inflammatory factors, and granule proteins or by affecting other immune cells function, promoting tumor cell proliferation, invasion, or metastasis; and (2) lymphocytes within the tumor microenvironment are a crucial component of the immune response. They can inhibit the proliferation and metastasis of tumor cells through various mechanisms, as well as induce apoptosis in these cells. The efficacy of anti‐tumor immunity largely depends on the quantity of lymphocytes present within the tumor microenvironment [13]; lymphocyte subsets, especially CD4^+^ T cells, to some extent reflect the immune status of GC patients, and a decrease in their number may contribute to tumor immune escape; (3) platelets are involved in inflammation and tumor progression. They wrap tumor cells in blood clots to protect them from lysis by natural killer cells. Procoagulant factors in tumor microenvironment of abnormal expression can induce platelet activation, leading to the release of growth factors by platelets and promoting tumor growth.

In conclusion, SII is negatively correlated with lymphocyte subset counts, with a decrease in CD4^+^ T cells identified as the primary factor influencing SII. This finding highlights the specific impairment of CD4^+^ T cell‐mediated immunity in the context of systemic inflammation. This finding highlights the impairment of CD4^+^ T cell‐mediated immunity within the context of systemic inflammation. Furthermore, elevated SII serves as an independent risk factor for poor prognosis. When compared to TNM staging, both SII and CD4^+^ T cell counts are non‐invasive and readily obtainable biomarkers. Collectively, these findings emphasize the prognostic significance of SII and its potential association with compromised immune function mediated by CD4^+^ T cells. This finding suggests that SII could serve as a potential biomarker for assessing both the immune status and prognosis of GC patients. However, there are some limitations to this study: First, as a single‐center retrospective study, this analysis may have inherent biases related to retrospective design and follow‐up compliance. Second, our retrospective dataset lacks treatment‐related variables, preventing subgroup analyses based on treatment modalities for GC patients. This limitation should be considered by readers when interpreting the study findings. Third, the sample size of this study is relatively small, necessitating further large‐scale prospective studies across multiple centers for validation.

## Author Contributions

**Xi Yan** and **Yinghao Niu:** conceptualization, methodology, visualization, writing – original draft; **Jing Du, Gang Lu, Jinyan Zhang,** and **Yiyang Guo:** data curation, investigation; **Yanyan Du, Xingxiao Yang,** and **Ming Ma:** funding acquisition, supervision, and writing – review and editing.

## Ethics Statement

The present study received ethics approval from the Fourth Hospital of Hebei Medical University (approval number: 2021KS037). All participants were provided with written informed consent.

## Consent

The authors have nothing to report.

## Conflicts of Interest

The authors declare no conflicts of interest.

## Supporting information

Supplementary Table 1.

Supplementary Table S1.

Supplementary Table S2.

## Data Availability

The datasets used and analyzed in the current study are available from the corresponding author upon reasonable request.
