# Supplementary material for: Correlation Between SII and Lymphocyte Subsets in Gastric Cancer Patients and Its Prognostic Importance
Source: Immun Inflamm Dis. 2026 Mar 23;14(3):e70399. doi: 10.1002/iid3.70399 (PMC13097496; doi:10.1002/iid3.70399)
Supplement: Supplementary file 1 — Supplementary Table 1. [file IID3-14-e70399-s003.docx]

Supplementary Table 1

| Variable | Schoenfeld Test P-value |
| --- | --- |
| TNM Staging | 0.905 |
| SII | 0.084 |
| CD4^+^ T cell | 0.382 |
| B cell | 0.330 |
| NK cell | 0.663 |
